# A streamlined approach for intelligent ship object detection using EL-YOLO algorithm

**DOI:** 10.1038/s41598-024-64225-y

**Published:** 2024-07-02

**Authors:** Defu Yang, Mahmud Iwan Solihin, Igi Ardiyanto, Yawen Zhao, Wei Li, Bingyu Cai, Chaoran Chen

**Affiliations:** 1 Faculty of Engineering, Technology and Built Environment, UCSI University, Kuala Lumpur, Malaysia; 2 Department of Electrical and Information Engineering, Faculty of Engineering, Universitas Gadjah Mada, Bulaksumur, Yogyakarta, 55281 Indonesia; 3 School of Computer Science and Technology (School of Software), Guangxi University of Science and Technology, Liuzhou, Guangxi China; 4 School of Advanced Manufacturing, Shantou Polytechnic, Shantou, China

**Keywords:** Intelligent ship, Lightweight YOLOv8, Object detection, Modified bounding box regression, Improved YOLOv8, Ocean sciences, Engineering

## Abstract

Maritime objects frequently exhibit low-quality and insufficient feature information, particularly in complex maritime environments characterized by challenges such as small objects, waves, and reflections. This situation poses significant challenges to the development of reliable object detection including the strategies of loss function and the feature understanding capabilities in common YOLOv8 (You Only Look Once) detectors. Furthermore, the widespread adoption and unmanned operation of intelligent ships have generated increasing demands on the computational efficiency and cost of object detection hardware, necessitating the development of more lightweight network architectures. This study proposes the EL-YOLO (Efficient Lightweight You Only Look Once) algorithm based on YOLOv8, designed specifically for intelligent ship object detection. EL-YOLO incorporates novel features, including adequate wise IoU (AWIoU) for improved bounding box regression, shortcut multi-fuse neck (SMFN) for a comprehensive analysis of features, and greedy-driven filter pruning (GDFP) to achieve a streamlined and lightweight network design. The findings of this study demonstrate notable advancements in both detection accuracy and lightweight characteristics across diverse maritime scenarios. EL-YOLO exhibits superior performance in intelligent ship object detection using RGB cameras, showcasing a significant improvement compared to standard YOLOv8 models.

## Introduction

Perceiving the surrounding environment is crucial for autonomous navigation in intelligent ships, as it provides foundational data for subsequent path planning and navigation safety assessments^1^. Currently, mature perception technologies heavily rely on the automatic identification system (AIS) and radio detection and ranging (RADAR). However, AIS requires all detected objects to have AIS systems installed^2^, relying on passive information perception from these objects, making it difficult to discern the authenticity of the input information. Additionally, RADAR technology has certain blind spots in object detection range and cannot obtain effective information, including contour, texture, and color, resulting in substantial limitations^3^. While these technologies are suitable for the current semi-automated state of autonomous navigation, they still impose limitations on achieving full autonomy. Presently, camera-based object perception technology, based on Red Green Blue (RGB) cameras, is a popular and extensively studied due to its ability to provide visual information at a relatively affordable price^4^.

**Figure 1:**
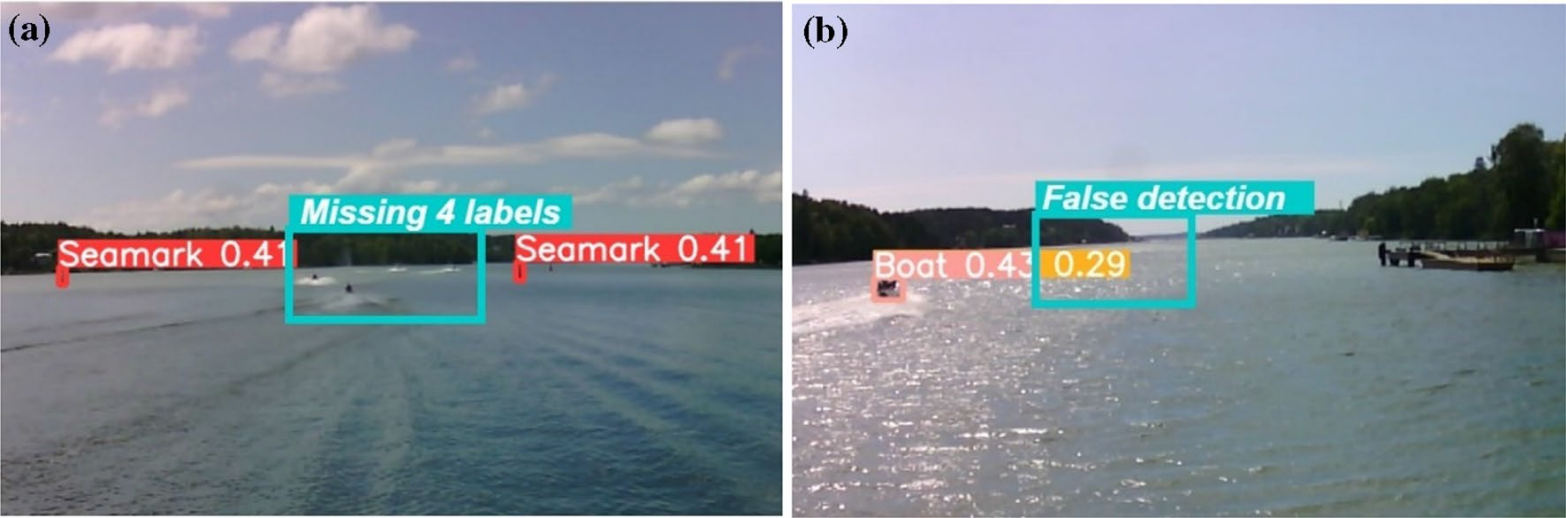
Typical examples of erroneous object detection in a typical maritime scene. (**a**) Missing detection, (**b**) False detection.

The main challenge of camera-based intelligent ship perception technology lies in processing and understanding the acquired images, identifying the categories and locating the objects, i.e. object detection^5^. Compared to land environments, maritime environments exhibit greater variability and factors of influence, such as waves, water surface refraction, virtual images, occlusions, and changing weather conditions^6^. Figure 1 illustrates exemplary instances of erroneous object detection resulting from the unique environmental conditions encountered in maritime settings. Figure 1a demonstrates a case of missed detections, where four vessels in the central region of the image were not properly identified, primarily due to the challenges posed by small target size and wave interference. Conversely, Fig. 1b exhibits instances of false positive detections, where objects that do not actually exist in the image have been erroneously identified, largely attributable to the confounding effects of reflections on the water surface and occlusion by nearby islands. These factors often lead to low-quality images resulting in poorer detection accuracy.

Moreover, high-performance hardware processing systems are often unfavorable for the widespread adoption of intelligent ships, such as those utilized in small vessels, commercial fishing boats, and unmanned ships. To address this, many scholars have explored the design of various embedded systems for deployment on vessels^7–9^ to enhance their intelligence capabilities. While it is difficult to quantify a single data point that definitively meets the requisite lightweight requirements, it is undeniable that the more lightweight the network model, the lower the hardware system demands, thereby better facilitating the development and proliferation of intelligent ship. However, many existing algorithms, although performing well in terms of detection accuracy, require larger parameter sizes and computational complexity. Achieving an effective balance between accuracy and computational complexity poses a challenge.

To address the aforementioned issues, this paper proposes a more efficient and lightweight network model called EL-YOLO for intelligent ship-based object detection in maritime environments. The model is based on the latest real-time model YOLOv8, with improvements made using the newly proposed AWIoU (adequate wise IoU), SMFN, and GDFP optimization techniques. Finally, to validate the proposed algorithm in a fair environment, comparative experiments are conducted on the SeaShips dataset^10^, representing a relatively simple environment, and the ABOships dataset^11^, representing a relatively complex environment. Additionally, ablation experiments and comparative analysis experiments using different loss functions are performed to further analyze the proposed algorithm. The results demonstrate that the proposed algorithm exhibits higher detection accuracy in intelligent ship object detection, while requiring significantly fewer computational parameters and costs.

The main contributions of this paper are summarized as follows:

1. The introduction of the novel AWIoU utilizes outlier degree to replace YOLOv8’s Complete IoU (CIoU) to reevaluate the performance of the BBR (bounding box regression) loss, and incorporates probability density to update the weights. This method can effectively avoid undue penalties on objects with low quality and achieve a fair distribution of gains for objects with varying qualities, thereby attaining higher detection accuracy. Comparative experiments clearly illustrate that AWIoU surpasses other loss functions such as CIoU^12^, SCYLLA IoU (SIoU)^13^, Efficient IoU (EIoU)^14^, and WIoU^15^ in detecting objects in maritime environments.
2. A novel methodology, known as SMFN, has been proposed to reconfigure the neck component, effectively enhancing the transmission and interpretation capabilities of low-quality object features to improve detection accuracy. This method primarily draws inspiration from shortcut connections, employing deep multi-fusion on features from different levels to enhance the understanding of relevant information. Low-level scale channels are also added at both the input and output ends to improve robustness against fluctuations at different scales.
3. A model compression approach, named GDFP, mainly establishes a mechanism of continuous elimination of filters based on information theory principles, where the importance score of filters is determined by the absolute value of the norm of input channels. By continuously removing the less important filters, the model can achieve an ideal lightweight configuration while maintaining the fundamental detection accuracy.
4. The proposal of EL-YOLO presents an efficient and lightweight network model tailored for maritime object detection. Building upon YOLOv8, EL-YOLO incorporates AWIoU, SMFN, and GDFP for upgrades. The proposed method effectively addresses the challenges of low accuracy and excessive model complexity encountered in maritime object detection.

The structure of this paper is as follows: Section “Introduction” introduces the research background, existing problems, and the proposed method for intelligent ship object detection. Section “Related work” provides an overview of the state-of-the-art research in the related fields. Section “Traditional approches” provides a detailed description of the proposed method. Section “Proposed method” presents the experimental setup including datasets, experimental environments and evaluation metrics. Section “Experimental setup” presents results, including comparative analysis of algorithms on different datasets and ablation experiments. Finally, Section “Results” presents the conclusion.

## Related work

### Traditional approches

The task of object detection in a maritime environment presents distinctive characteristics in terms of region of interest (ROI), with the sea-sky line (SSL) being notably significant. Various techniques are employed for determining ROI, and traditional approaches involve several key procedures, including preprocessing^16^, feature extraction^17^, and object classification^18^. These procedures bear similarities to their land-based object detection counterparts. Numerous scholars have concentrated on the rapid identification of targets through the determination of the sea-sky line (SSL). Ma et al.^19^ applied the least square method to initially select the SSL, optimizing candidate lines through non-linear suppression to derive the final SSL for precise object localization. In a similar vein, Xin et al.^20^ employed the Hough Transform to fit lines onto preprocessed images. Their approach involved multiple experiments to select various thresholds, enabling them to compare and filter the fitted SSL for a closer adherence to the actual lines. Additionally, alternative methods for SSL determination encompass the radon transform^21^ and random sampling consistency^22,23^.

Dynamic object extraction stands out as a key research focus in the extraction of regions of interest (ROI) for maritime objects. By scrutinizing the movement of ships and floating objects, researchers can swiftly discern distinctions between these objects and the background. Yang et al.^24^ introduced an adaptive inter-frame difference method tailored for detecting slowly moving ship regions. This method employs frame-to-frame image differencing, mitigating the influence of environmental factors.

Another approach, the background difference method, shares similarities but takes a different route. Instead of directly extracting differences between frames, it first establishes the background and then subtracts it to pinpoint the ROI. For example, Xiao et al.^25^ leveraged the visual background extractor to extract and continuously update background information, enhancing detection performance, particularly for smaller distant objects, by incorporating more background parameter information. Different techniques, including the utilization of the optical flow method^26^, have been employed. To sum up, conventional approaches for maritime object detection provide simplicity, low computational complexity, and excellent real-time performance. Nevertheless, their constraints involve a lack of robustness and the requirement for adjustments in diverse environments, presenting difficulties in effectively responding to intricate changes in maritime surroundings.

### Deep learning approches

With the continuous enhancement of computer hardware capabilities, the adoption of deep learning in maritime object detection has significantly expanded since 2012. The pivotal factors influencing deep learning methods are the datasets and network models. presently, publicly available datasets for maritime object detection are somewhat limited, including McShips^27^, SMD^28^, Seaships, ABOships, among others. The quality of the dataset plays a crucial role in influencing detection performance^29^. The accuracy and speed of the same network model can vary when applied to different environmental datasets.

Another critical aspect is the network model, with contemporary maritime object detection primarily relying on convolutional neural network (CNN) models. These models can be categorized as anchor-free or anchor-based, depending on the requirement for anchors. anchor-free approaches, characterized by the absence of anchor settings, offer simpler calculations and include methods such as fully convolutional one-stage object detection (FCOS)^30^ and Reppoints^31^. However, anchor-free methods often exhibit suboptimal performance in terms of detection accuracy.

Currently, the mainstream maritime object detection network models are anchor-based, utilizing anchor boxes for initial selection, and subsequently determining the final object classification and localization. Based on the number of corrections, anchor-based methods can be categorized as one-stage models or two-stage models, corresponding to one or two rounds of correction, respectively. In 2019, Qi et al.^32^ proposed an improved two-stage model, Faster R-CNN, for ship detection. It enhances the comprehension of valuable information through down-sampling and optimizes the parameters between the main network and sub-network to improve object detection accuracy. In 2018, Nie et al.^33^ introduced an enhanced mask R-CNN model for shore-based ship detection management. Mask R-CNN belongs to another mainstream two-stage model, and the improved algorithm incorporates soft-non-maximum suppression to enhance environmental adaptability. In 2019, Jin et al.^34^ utilized an improved single shot multibox detector (SSD) algorithm for detecting distant small maritime objects. SSD is a widely adopted one-stage model, and the enhanced algorithm incorporates a squeeze-and-excitation network module to improve feature extraction capabilities. Yutong et al.^35^ presented a hierarchical deep learning framework combining a CNN, Extreme learning machine, and a fuzzy slime mould optimizer for real-time sonar image recognition, demonstrating excellent detection accuracy. Kamalipour et al.^36^ proposed a novel deep convolutional-recurrent autoencoder for robust passive ship detection and classification in complex underwater acoustic environments. Tian et al.^37^ propose a Radial basis function neural network enhanced by the chimp optimization algorithm for improved underwater image detection and recognition. Najibzadeh et al.^38^ propose a CNN model enhanced by a robust Grey Wolf optimizer for active sonar image classification and achieved satisfactory experimental results in terms of detection accuracy and computational complexity.

Despite the demonstrated improvements in detection accuracy and robustness with deep learning methods compared to traditional approaches, it‘s important to note that they often require additional detection time and may not always meet the real-time detection requirements of intelligent ships.

### YOLO algorithm

The YOLO algorithm series is a classic example of a one-stage model that has consistently aimed to strike a balance between detection accuracy and real-time performance^39^. Due to its advantage in real-time processing, this series has found wider applications in practical object detection tasks, particularly in scenarios that demand real-time requirements, such as real-time object search for unmanned aerial vehicles^40^ and object detection for unmanned vessels. For instance, He et al.^41^ employed an improved version of YOLOv4 to detect ships approaching harbor berths. Their approach enhanced the input data clustering using the k-means algorithm, thereby improving the detection accuracy. Yang et al.^42^ proposed an object detection algorithm for unmanned vessels, which incorporated the ShuffleNetv2 attention mechanism into YOLOv5n to enhance feature extraction capabilities. They also utilized the add fusion method to strengthen information comprehension, thereby improving the detection performance. In another study, Zhao et al.^43^ presented a detection algorithm for maritime personnel search and rescue. They made improvements to YOLOv7 by introducing the parameter-free SimAM for efficient target search region localization and incorporating an additional small object detection head to enhance the detection capability for small objects. While the YOLO series has undergone continuous improvements and demonstrated commendable detection performance, there is still room for enhancing detection accuracy, particularly in complex maritime environments characterized by factors such as waves, light reflections, and the presence of far small objects. Furthermore, as more embedded devices become common and cost control becomes crucial, there's a growing need for lightweight network models. Balancing these two aspects in complex maritime environments has become a new research challenge.

The YOLO architecture typically consists of three key components: the backbone, neck, and head. The backbone extracts feature information at various scales, and the neck integrates and interprets data from these scales. The head is responsible for localization and classification tasks. Notably, the neck component plays a crucial role in information interpretation. The curent version of YOLO algoritm is YOLOv8. Before YOLOv4, the neck primarily utilized the FPN (feature pyramid network) structure, enabling multi-scale fusion. Subsequently, the adoption of the PAN-FPN (path aggregation network with feature pyramid network) structure aimed at achieving more comprehensive fusion but resulted in a deeper network architecture. However, these structures are susceptible to the loss of low-quality object information during transmission, particularly in complex maritime environments where low-quality objects are prevalent. Consequently, the advantages in detection accuracy of this specific neck architecture are not as pronounced in maritime settings.

## Proposed method

Due to the satisfactory performance of the YOLO series in terms of real-time processing and detection accuracy^44^, this study proposes an improved algorithm named EL-YOLO, built upon the latest and most advanced YOLOv8 algorithm^45^. The specific ideas of the proposed algorithm are, focusing on AWIoU, SMFN, and GDFP, as follows:

1. Concerning the evaluation of object localization loss, the proposed method adopts AWIoU to replace CIoU in YOLOv8n. AWIoU is a novel loss function that intelligently assigns different gains to examples of varying qualities. This approach mitigates adverse gradients associated with low-quality examples, guiding the BBR process in a more reasonable and efficient manner.
2. For the neck part, modifications involve the introduction of SMFN for deep information analysis. Initially, additional interfaces are incorporated on the backbone to mitigate information loss during long-distance transmission. Subsequently, a multi-feature fusion approach is employed using shortcut skip connections, facilitating a profound understanding of the features. Finally, a low-level scale is added to the output end to enhance the detection capability for distant small objects.
3. To achieve a more lightweight design, we employed GDFP to rearrange the weights and greedily remove unnecessary filters to compress the network structure.

**Figure 2:**
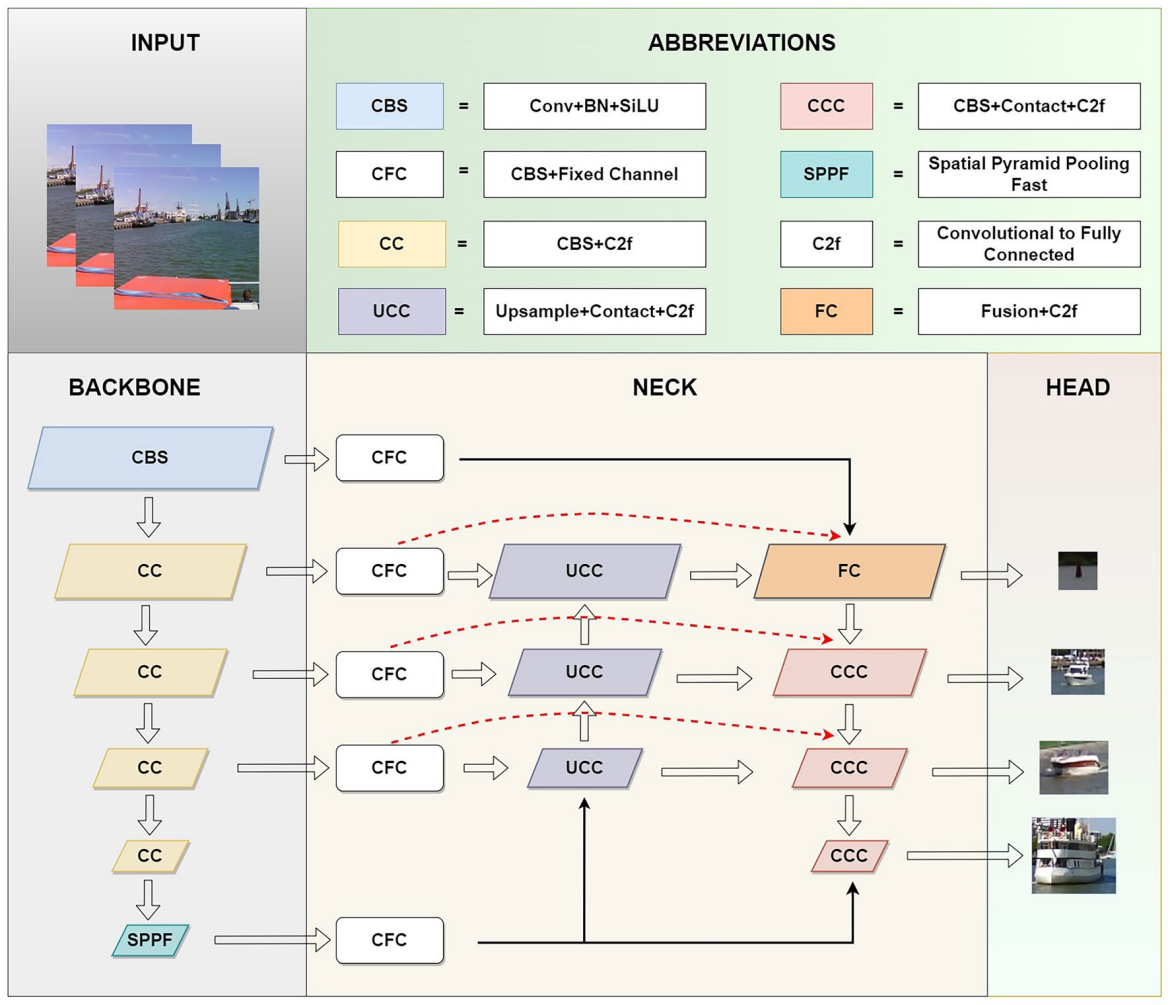
The main network architecture of EL-YOLO (*SPPF* spatial pyramid pooling fast, *C2f* convolutional to fully connected).

The detailed main network structure of EL-YOLO is illustrated in Fig. 2, and the subsequent sections will delve into discussing the specific ideas behind the improvement.

### AWIoU (adequate wise IoU)

The two fundamental tasks in object detection are object classification and localization. Assessing classification accuracy is relatively straightforward, involving a simple comparison between the predicted class and the ground truth. Localization, on the other hand, is typically assessed using intersection over union ($IoU$)^46^. Assuming the predicted bounding box is denoted as ${B}_{p}$ and the actual detection box as ${B}_{r}$, the formula for IoU, as shown in Eq. (1), is used:

1 $$IoU=\frac{{B}_{P}\cap {B}_{r}}{{B}_{P}\cup {B}_{r}}.$$

When ${B}_{p}$ and ${B}_{r}$ have no intersection, $IoU$ is 0, and when they perfectly overlap, IoU is 1. The range of $IoU$ values is between 0 and 1, with higher values indicating greater proximity between the predicted and real boxes. Like other methods, YOLO structures often employ IoU as a loss function^47^ to dynamically adjust parameters in subsequent iterations, with the goal of increasing $IoU$ towards 1 or a set threshold. Therefore, the choice of the loss function is crucial, as it influences the convergence speed and quality of IoU. The initial IoU loss function, denoted as ${\mathcal{L}}_{IoU}$ and shown in Eq. (2), is as follows:

2 $${\mathcal{L}}_{IoU}=1-\frac{{B}_{P}\cap {B}_{r}}{{B}_{P}\cup {B}_{r}}.$$

However, in practical applications, this basic loss function often yields unsatisfactory results. While the overlap area between the predicted box and the real box is acknowledged as a crucial factor, it is not the sole determinant. Other factors, such as the distance between the two boxes, aspect ratio, and angle, can also impact the convergence speed and effectiveness of the detection box. Therefore, YOLOv8 adopts the complete intersection over union (CIoU) to evaluate the loss, as shown in Eq. (3)^12^:

3 $${\mathcal{L}}_{CIoU}=1-\frac{{B}_{p}\cap {B}_{r}}{{B}_{p}\cup {B}_{r}}+\frac{{\rho }^{2}\left({b}_{p},{b}_{r}\right)}{{c}^{2}}+av.$$

Here, ${b}_{p}$ and ${b}_{r}$ represent the centers of the predicted box and the real box, respectively. ${\rho }^{2}\left({b}_{p},{b}_{r}\right)$ denotes the Euclidean distance between the centers of the two boxes, c is the diagonal distance of the minimum enclosing box, and $av$ is the penalty term for aspect ratio. CIoU performs well in land situations, especially in indoor environments. However, it underperforms in marine environments, particularly during adverse weather conditions encountered during intelligent ship navigation. This is primarily due to two reasons:

1. In marine environments, especially in harsh conditions, a considerable portion of the generated examples are of low quality. CIoU adds penalties for aspect ratio and distance, which have a significant convergence effect on high-quality examples but often overly penalize low-quality ones.
2. In general, intelligent ship object detection involves the presence of simultaneous low-quality and high-quality samples. Applying the same penalty criterion to both types poses a challenge, as it either lacks sufficient penalty for high-quality examples or tends to excessively penalize low-quality ones.

To address the first issue, Tong et al.^15^ proposed WIoUv1 (wise-IoU version 1) to adjust the penalty appropriately, as shown in Eq. (4):

4 $${\mathcal{L}}_{WIoUv1}=\left(1-\frac{{B}_{p}\cap {B}_{r}}{{B}_{p}\cup {B}_{r}}\right)\text{exp}\left(\frac{{\rho }^{2}\left({b}_{p},{b}_{r}\right)}{{c}^{2}}\right).$$

In addition to the overlap area, the distance between centers emerges as a pivotal factor influencing IoU convergence. Therefore, WIoUv1 removes the aspect ratio penalty term $av$ from CIoU and transforms the penalty term $\frac{{\rho }^{2}\left({b}_{p},{b}_{r}\right)}{{c}^{2}}$ into an exponential form to alleviate overfitting and make the predicted box more cautious during movement.

To address the second issue, Tong et al.^15^ proposed WIoUv2 and WIoUv3 to achieve wise gain allocation, providing different gains to low-quality and high-quality examples. Both algorithms build upon WIoUv1 by incorporating dynamic focal mechanisms. They also demonstrated that WIoUv3 performs better than WIoUv2 through experiments. Thus, the focus will be on discussing WIoUv3, as shown in Eq. (5):

5 $${\mathcal{L}}_{WIoUv3}=\left(1-\frac{{B}_{p}\cap {B}_{r}}{{B}_{p}\cup {B}_{r}}\right)\text{exp}\left(\frac{{\rho }^{2}\left({b}_{p},{b}_{r}\right)}{{c}^{2}}\right)\frac{\frac{{\mathcal{L}}_{IoU}^{*}}{\overline{{\mathcal{L} }_{IoU}}}}{\delta {\alpha }^{\frac{{\mathcal{L}}_{IoU}^{*}}{\overline{{\mathcal{L} }_{IoU}}}-\delta }}.$$

In this equation, ${\mathcal{L}}_{IoU}^{*}$ represents the current separable calculation of ${\mathcal{L}}_{IoU}$, while $\overline{{\mathcal{L} }_{IoU}}$ denotes the average ${\mathcal{L}}_{IoU}$. Tong et al.^15^ have empirically demonstrated that when the hyperparameters α = 1.9 and δ = 3, ${\mathcal{L}}_{WIoUv3}$ achieves the best performance. Therefore, the key lies in determining $\overline{{\mathcal{L} }_{IoU}}$. When considering $\overline{{\mathcal{L} }_{IoU}}$ in ${\mathcal{L}}_{WIoUv3}$, it only takes a small constant m into account to accelerate convergence. However, in practical experiments, its effectiveness for maritime object detection is not significant. The underlying reason is the failure to fully utilize the dynamic focal mechanism.

To address this issue, this study proposes AWIoU based on WIoUv3 to enable adequate wise gain allocation. The proposed AWIoU is expressed in the following Eq. (6):

6 $${\mathcal{L}}_{AWIoU}\left(t\right)=\left(1-\frac{{B}_{p}\cap {B}_{r}}{{B}_{p}\cup {B}_{r}}\right)\text{exp}\left(\frac{{\rho }^{2}\left({b}_{p},{b}_{r}\right)}{{c}^{2}}\right)\frac{\frac{{\mathcal{L}}_{IoU}^{*}}{\left\{\left(1-g\right)\overline{{\mathcal{L} }_{IoU}\left(t-1\right)}+g{\mathcal{L}}_{IoU}^{*}\right\}}}{\delta {\alpha }^{\frac{{\mathcal{L}}_{IoU}^{*}}{\left\{\left(1-g\right)\overline{{\mathcal{L} }_{IoU}\left(t-1\right)}+g{\mathcal{L}}_{IoU}^{*}\right\}}-\delta }}.$$

The main focus is to adequately consider $\overline{{\mathcal{L} }_{IoU}}$ in Eq. (5) by introducing the probability density $g$ to adaptively adjust the updating weights for the average value. This dynamic tracking of loss variations enables a more refined gain allocation strategy for different quality examples, leading to an adequate wise allocation approach.

### SMFN (short multi-fuse neck)

**Figure 3:**
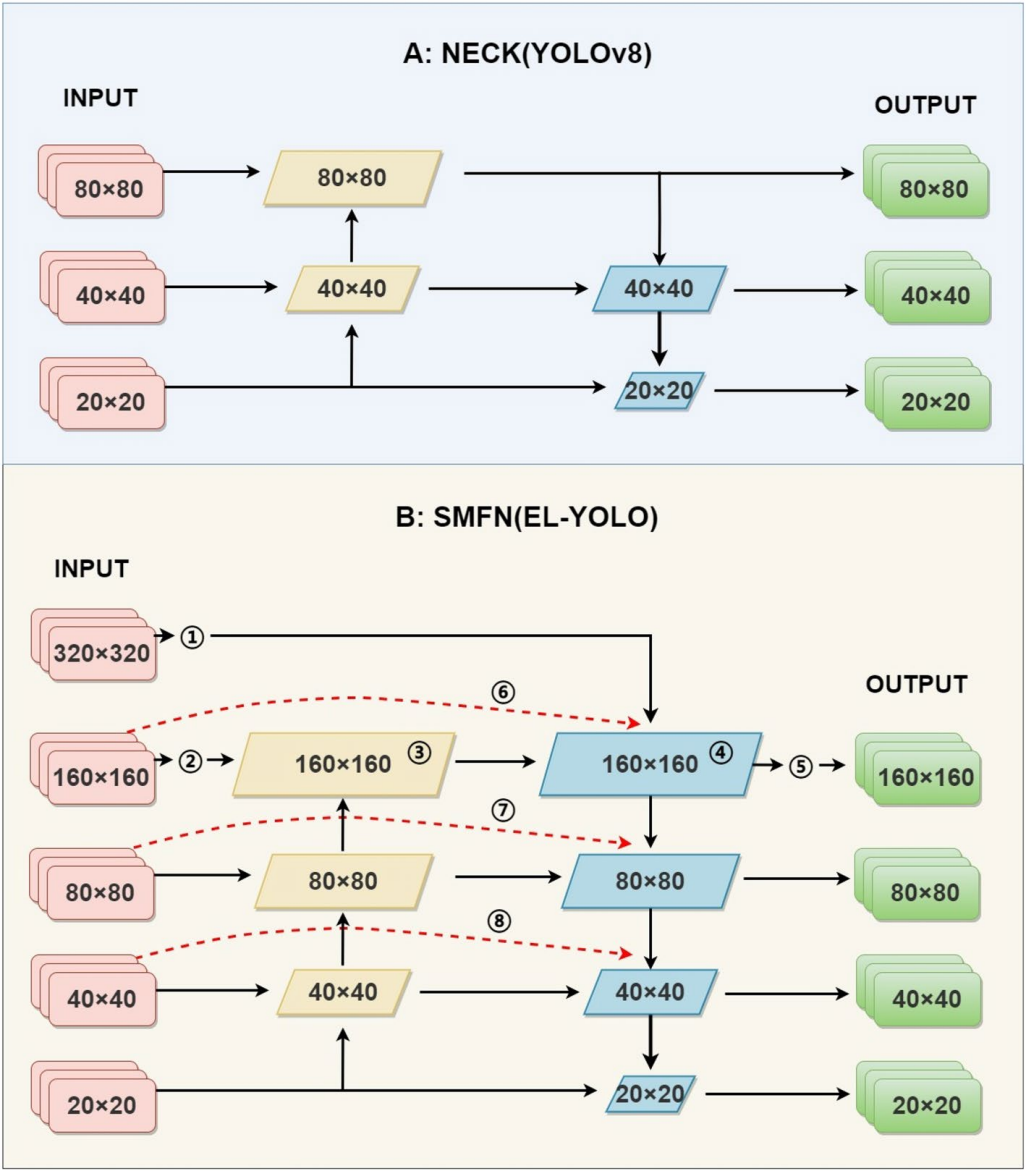
The neck part structure of YOLOv8 and EL-YOLO.

The YOLO architecture is composed of three fundamental components: the backbone, neck, and head. The neck part plays a crucial role in analyzing the extracted feature information from the backbone in the context of object detection. In YOLOv8, the neck module operates on three scales: 80 × 80, 40 × 40, and 20 × 20. As depicted in the upper part of Fig. 3 (A: NECK(YOLOv8)), it utilizes two complementary pyramid structures to parse the features. While this design yields favorable results in general indoor land situations, it often falls short when detecting objects in maritime environments. This limitation can be attributed to two main factors:

Firstly, maritime environment are inherently more complex than land one, making it more challenging to interpret extracted information due to the presence of phenomena like ghosting, reflections, and occlusion by fog. Consequently, understanding the extracted information becomes more difficult, and the loss of information during long-distance transmission has a more pronounced impact.

Secondly, in intelligent ship maritime object detection, a significant focus is placed on detecting smaller objects compared to terrestrial vehicle detection. This emphasis arises from the challenges ships face in maneuvering, requiring extended warning distances. This necessitates a broader scope of analysis to effectively detect these diminutive objects.

In this regard, this study proposes a new neck part called SMFN, as illustrated in the part B of Fig. 3, i.e. B: SMFN(EL-YOLO). Firstly, while keeping the structure of the original backbone unchanged, SMFN fully considers different scales during the backbone extraction process by incorporating feature information from two larger scales, namely 320 × 320 and 160 × 160, denoted as ① and ②. This approach not only provides additional information input to the existing parsing structure but also mitigates the impact of insufficient effective features in low-quality examples. Moreover, it allows the primary-level feature information to directly influence the information fusion in ④, alleviating the loss of information during the information transmission process.

Furthermore, to achieve more precise understanding of the feature information for smaller objects, this study adds two 160 × 160 parsing scales, denoted as ③ and ④, to the original neck component. Correspondingly, a new scale, denoted as ⑤, is introduced for improved detection of small objects, particularly those recently appearing on the SSL. This enhancement aims to provide more effective parsing information for small object detection. Finally, drawing inspiration from the concept of shortcut connections, this study employs three dotted red lines, represented by ⑥, ⑦, and ⑧, to establish skip connections from the initial input of 160 × 160, 80 × 80, and 40 × 40 feature information of the backbone to the final stage of parsing. This effectively mitigates the issue of feature loss during the information transmission process at these three scales. Additionally, the deep fusion of multiple types of information serves as an effective method for in-depth parsing, enhancing the ability to comprehend information features, especially in cases where high-quality information features are unavailable for certain examples.

### GDFP (Greedy-driven filter pruning)

Network pruning is a commonly-adopted approach for model compression^48^, aiming to remove redundant weights while minimally impacting the detection accuracy, thereby slimming down the network. Our method is based on the Pruning Filters technique proposed by Li et al.^49^, as the redundancy of filters increases significantly with the growing network depth. By selectively removing less important filters, the network structure can be significantly reduced.

**Figure 4:**
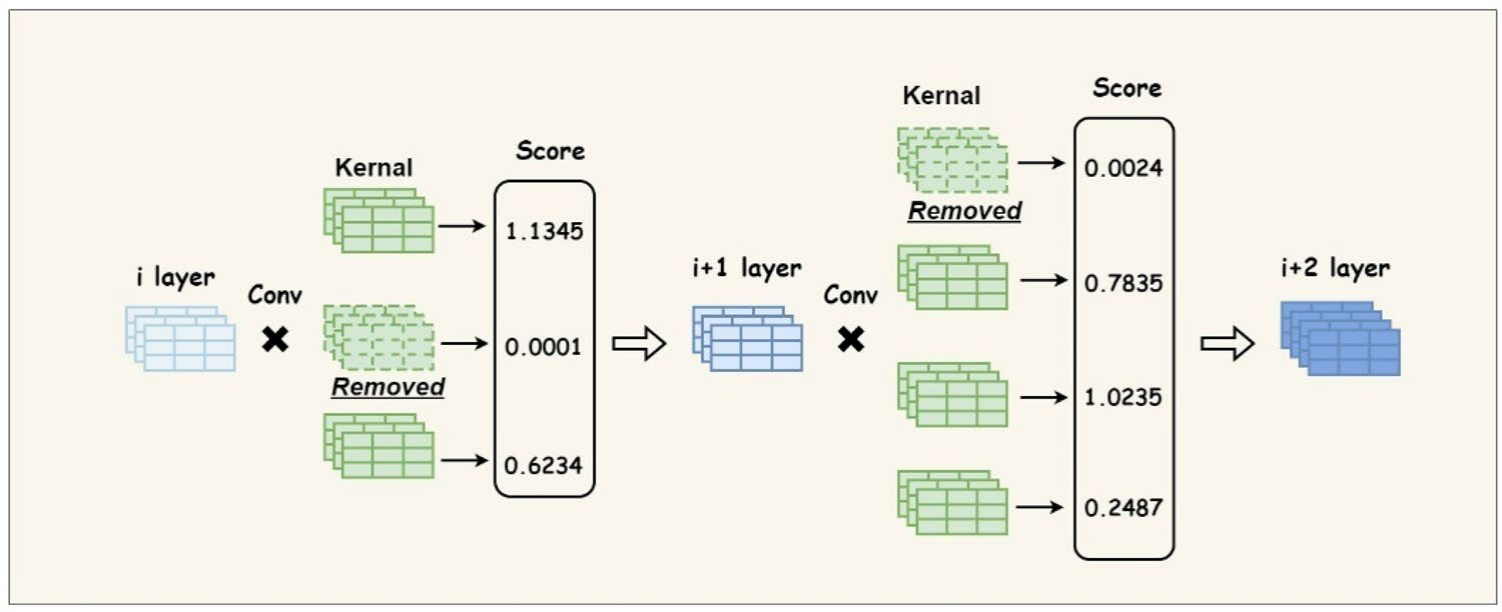
The layer-wise greedy filter pruning example.

The overall approach bears similarity to optimal brain damage^50^, wherein we leverage information-theoretic principles to greedily remove the least important filters. The pruning process is carried out on a per-layer basis, where the importance score of each filter is computed, and then the filters with the lowest scores are removed in a greedy fashion, as illustrated in Fig. 4. This greedy behavior continues until the desired compression ratio is achieved. Specifically, the filters with the lowest scores in each layer are successively removed, followed by the second-lowest, and so on, until the target compression is met.

Regarding the calculation of the importance score, the proposed approach utilizes the L1-norm of the filters, as shown in Eq. (7):

7 $${S}_{i,j}={\Vert {F}_{i,j}\Vert }_{1}=\sum \left|{F}_{i,j}^{n}\right|.$$

Here, F_i,j_ represents the j-th convolutional kernel in the i-th layer, and ${S}_{i,j}$ denotes the score of that particular kernel. The variable n denotes the number of input channels. Specifically, for the j-th convolutional kernel, the absolute values of the L1-norms of the kernels across all input channels are summed to obtain the final importance score of that filter.

**Figure 5:**
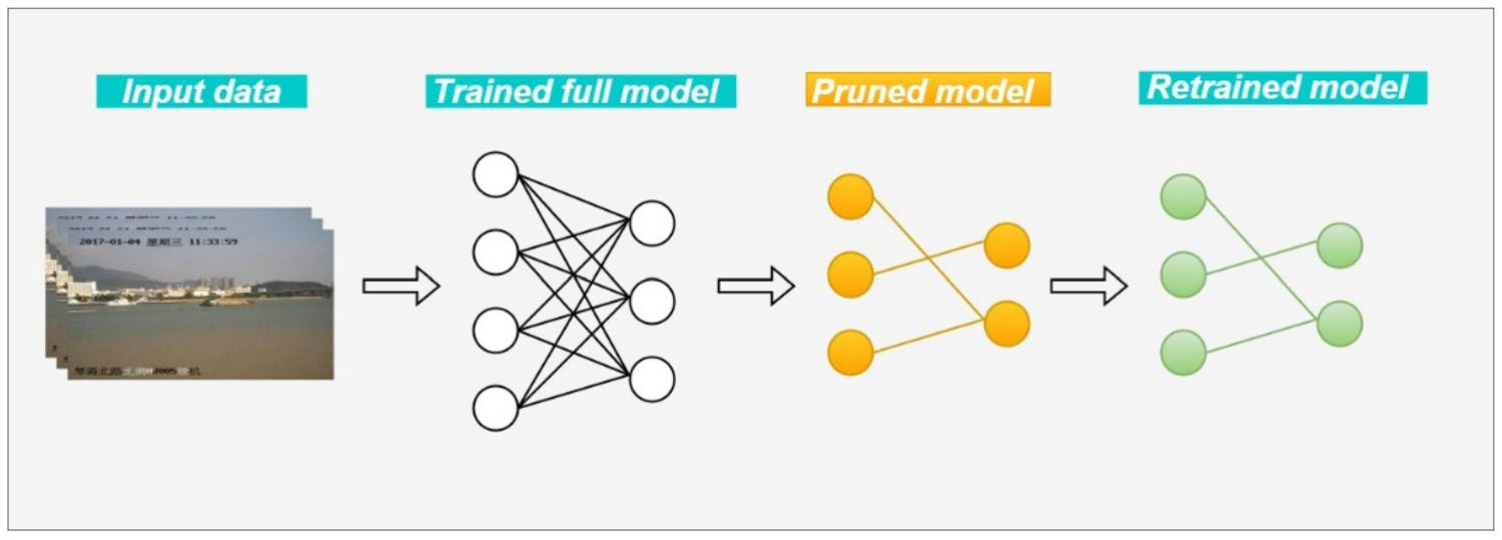
EL-YOLO pruning workflow.

As illustrated in Fig. 5, the overall pruning process can be summarized as follows:First, a trained full model is obtained by training the model architecture enhanced as per the improvements detailed in Sects. “AWIoU (Adequate Wise IoU)” and “SMFN (short multi-fuse neck)” .Subsequently, the importance scores of the filters in each layer are computed and ranked. Based on the target compression ratio, a portion of the filters with the lowest scores are greedily removed, resulting in the creation of a pruned model with a modified network structure. Undeniably, the detection accuracy of the model tends to deteriorate significantly after the pruning process. Therefore, the final step involves retraining the pruned model to recover its original performance level.

The complete algorithmic implementation of this process is depicted in Algorithm 1.

**Algorithm 1:**
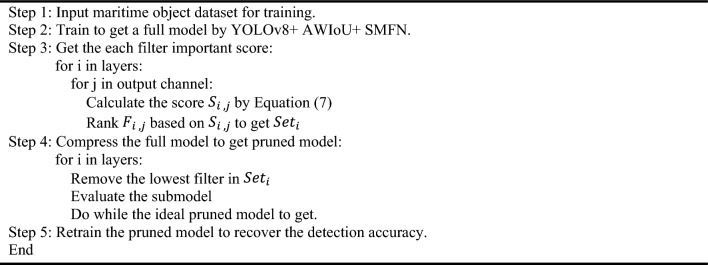
Greedy-driven filter pruning.

## Experimental setup

### Datasets

This article aims to conduct a set of empirical investigations employing the proposed methodologies, utilizing two publicly accessible datasets. The first dataset, named ‘Seaships’, specifically designed for a simplified environmental context and is intended for comparative experiments with mainstream detection models. It can be accessed at http://www.lmars.whu.edu.cn/prof_web/shaozhenfeng/datasets/SeaShips%287000%29.zip. The second dataset involves a more intricate environmental setting and primarily utilizes the ABOships dataset (https://zenodo.org/records/4736931), serving as the primary dataset throughout the subsequent experiment unless otherwise specified.

**Figure 6:**
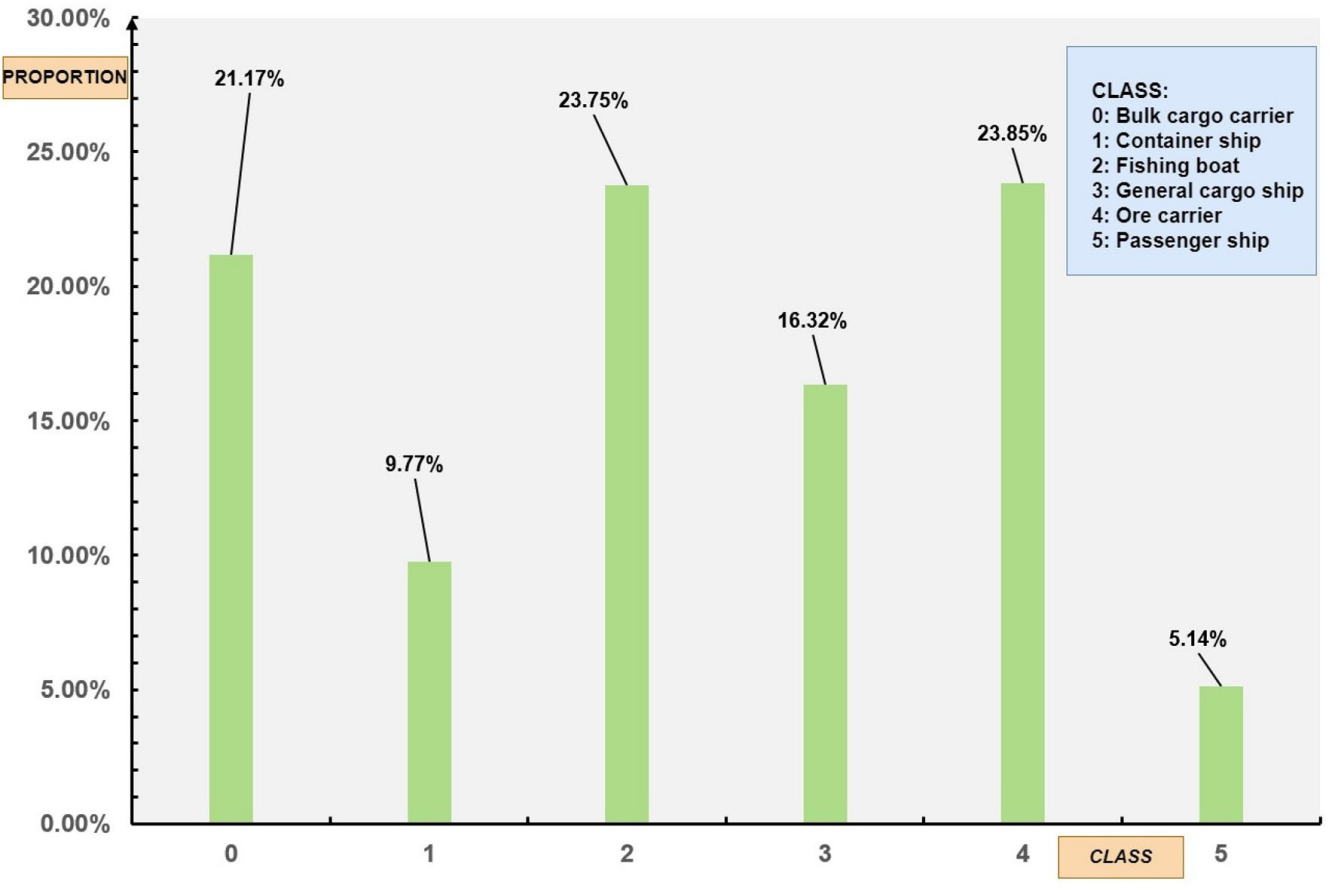
Distribution of objects in Seaships dataset.

The Seaships dataset contains a total of 7000 images with 9221 detection objects. On average, each image includes 1.32 objects. The detailed distribution of each object category is shown in Fig. 6. This dataset is relatively simple in terms of the detection task. It is captured from fixed camera positions at the port, providing a wide field of view. It can simulate the object detection of intelligent ships docking at the port. The detection environment is relatively straightforward.

The ABOships dataset consists of 9041 images with 41,967 detection objects. On average, each image contains 4.64 detection objects. The detection task in this dataset is relatively complex, and the camera positions are fixed on the ships. The dataset is collected during the normal navigation process of ships, simulating the object detection of intelligent ships during regular navigation. It is prone to occlusions, reflections, and adverse weather conditions, making the situation relatively complex.

**Figure 7:**
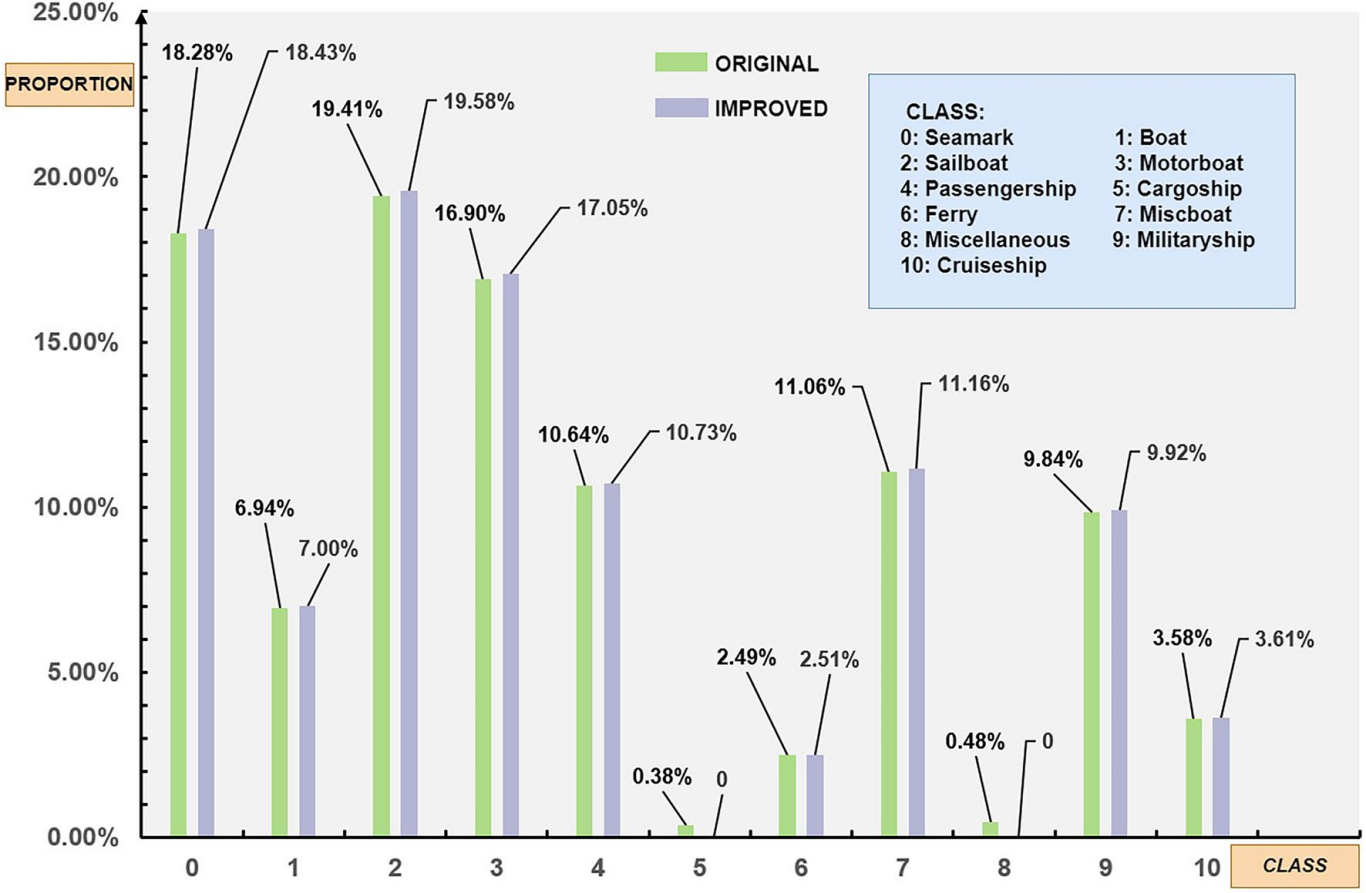
Distribution of objects before and after optimization for ABOships dataset.

The ABOships dataset consists of 11 categories, and the distribution of each category is shown in the green bar graph in Fig. 7. Class 5 and Class 8 have less than 0.5% proportion, indicating a severe class imbalance compared to other categories. Even with data augmentation, it is challenging to address this imbalance. Iancu et al.^51^, also acknowledged this issue but chose to merge categories, which contradicts the original intention of fine-grained object detection and can confuse the detection network. Therefore, this article adopts an exclusion method, removing these two categories that account for a total of 0.82% proportion. The updated distribution of the remaining categories is shown in the purple bar graph in Fig. 7. Although there is a slight increase in their representation, the overall impact is minimal. The resulting new dataset is referred to as ABOships-9.

### Training strategy

The YOLOv8 model has five versions^45^ namely: YOLOv8n, YOLOv8s, YOLOv8m, YOLOv8l, and YOLOv8x. Apart from differences in the network depth and width, the network features used are the same. Therefore, the optimized model EL-YOLO, derived from YOLOv8, also has corresponding versions: EL-YOLOn, EL-YOLOs, EL-YOLOm, EL-YOLOl, and EL-YOLOx. In theory, experiments can be conducted with any of these versions. However, considering the limitations of computational resources, this study primarily focuses on the smallest version of the model, EL-YOLOn.

During the experiments, the stochastic gradient descent (SGD) optimizer was employed to update and converge the parameters. This optimization method is advantageous for solving non-convex problems and helps escape local optima to search for global optima. The dataset was divided according to the following strategy: 70% for training, 10% for validation, and 20% for testing. See Table 1 for more details on the experimental key parameters.

**Table 1:** Experimental key parameters.

Category	Parameters	Result
Training parameters	Optimizer	SGD
	Batch size	4
	Epochs	200
	Pretrain	Closed
	Learning rate	0.01
	Input images size	640 × 640
	Momentum	0.937
	Weight_decay	0.0005
	Warmup_epochs	3.0
Experimental environment parameters	CPU	i7-12700H
	GPU	NVIDIA GeForce RTX 3060
	GPU memory size	6 GB
	Programming language	Python 3.9.0
	Operation system	Win 11
	Module platform	Pytorch1.13.0 + cuda11.6

### Evaluation metrics

This paper conducts a thorough evaluation of the proposed model, examining it from two perspectives: detection accuracy and efficiency in terms of lightweight design. The evaluation of detection accuracy is described using Precision and Recall, both respectively expressed in Eqs. (8) and (9)^52^.

8 $$\text{Precision}=\frac{TP}{TP+FP},$$

9 $$\text{Recall}=\frac{TP}{TP+FN}.$$

Here, TP refers to true positive, indicating instances that are correctly detected as positive. FP represents false positive, denoting instances that are incorrectly detected as positive when they are actually negative. FN represents false negative, indicating instances that are incorrectly detected as negative when they are actually positive. Since object detection tasks involve both object classification and localization, a positive detection must satisfy both the positive classification and positive localization criteria. Otherwise, it should be considered as a negative detection. The positivity of classification is straightforward, while the positivity of localization is primarily determined by the IoU, as stated in Eq. (1), where an IoU value exceeding a certain threshold indicates positive localization.

In principle, aiming for higher values of precision and recall is ideal. However, in real-world situations, these two metrics frequently clash, posing a challenge in intuitively comparing detection accuracy. Therefore, the detection task employs a more comprehensive evaluation metric called mean average precision (mAP), as shown in Eq. (10).

10 $$mAP=\frac{\sum_{i=1}^{m}A{P}_{i}}{m}.$$

Here, $m$ represents the number of object categories to be detected, and $AP$ denotes the average precision, which quantifies the area under the precision-recall curve. The calculation of $AP$ is detailed in Eq. (11)^53^.

11 $$AP=\sum_{i=1}^{n-1}\left({R}_{i+1}-{R}_{i}\right)\underset{{R}_{i}\le R\le {R}_{i+1}}{\text{max}}P\left({R}_{i}\right).$$

This equation calculates the area enclosed by selecting several points on the curve. In the equation, $n$ is the total number of points on the curve to be selected, ${R}_{i}$ represents the horizontal coordinate of the i-th point, and $P({R}_{i})$ represents the value on the curve at that point.

The specific detection accuracy metrics employed in this study are mAP0.5 and mAP0.5:mAP0.95. The metric mAP0.5 indicates that when the IoU value exceeds 0.5, the object is considered positively located, and the mAP value is computed accordingly. On the other hand, mAP0.95 requires a higher overlap between the detected and the real bounding box, with an IoU threshold of 0.95 for positive detection. Therefore, mAP0.5:mAP0.95 provides a stringent evaluation of detection accuracy, suitable for scenarios with higher safety requirements during maritime navigation.

Regarding the lightweight design aspect, this study adopts the parameter count (PC), frames per second (FPS) and floating point operations (FLOPs) to evaluate the model ‘s efficiency. PC as defined in Eq. (12)^54^.

12 $$\text{PC}=\left({C}_{\text{in}}{K}^{2}+\updelta \left(\text{bias}\right)\right)\text{M}{C}_{out},bias\in (\text{0,1})$$

Here, ${C}_{\text{in}}$ represents the number of input channels, ${C}_{out}$ is the number of output channels, $K$ denotes the size of the convolutional kernel, $bias$ represents the bias term, and $M$ is the number of convolutional kernels. FLOPs as defined in Eqs. (13), (14)^4^.

13 $$\text{FLOPs}\left(\text{con}\right)=\left(2{C}_{\text{in}}{K}^{2}- 1+\updelta \left(\text{bias}\right)\right)\text{S}{C}_{out},bias\in (\text{0,1})$$

14 $$\text{FLOPs}\left(\text{full}\right)=\left(2\text{I}-1+\updelta \left(\text{bias}\right)\right)\text{O},bias\in (\text{0,1})$$

Here, FLOPs(con) and FLOPs(full) represent the distinct counts of floating-point operations in convolutional connections and fully connected layers, respectively. S denotes the spatial area of the output layer, I specifies the number of input layers, and O corresponds to the number of output layers.

As illustrated in Eq. (15), the FPS can be calculated as follows:

15 $$FPS=\frac{1000}{preprocess+inference +postprocess}.$$

Here, preprocess, inference, and postprocess denote the duration of these respective stages, measured in milliseconds (ms).

## Results

### Comparative analysis of AWIoU performance against mainstream loss functions

To assess the impact of the proposed AWIoU on maritime object detection, we employed YOLOv8n as the base network architecture and compared AWIoU with the current mainstream loss functions, namely CIoU, EIoU, SIoU, and WIoU. For EIoU and SIoU, this experiment chose the focal mechanism exhibiting superior performance, referred to as EIoU(F) and SIoU(F) respectively. As for WIoU, this experiment uses the v3 version which demonstrated the best performance, and input the recommended hyperparameters α = 1.9 and δ = 3 into the experiments. The test results are presented in Table 2.

**Table 2:** Comparation of detection accuracy of AWIoU against mainstream loss functions.

Base model	Loss function	Metrics	
		mAP0.5	mAP0.5:0.95
YOLOv8n	CIoU	0.623	0.318
	SIoU (F)	0.62	0.316
	EIoU (F)	0.625	0.319
	WIoUv3	0.628	0.32
	AWIoU	**0.635**	**0.321**

Within the tabular data, the key metrics exhibiting the optimal performance are bolded; presently, the superior indicators are all manifested within the AWIoU loss function.

SIoU(F), due to the addition of numerous constraints^13^, such as angle loss and shape loss, severely hindered the regression of low-quality samples, resulting in the least desirable performance. Both EIoU(F) and WIoUv3 exhibited varying degrees of improvement compared to CIoU. However, their performance enhancement was limited by the lack of genuine release of the focal mechanism's potential. The proposed AWIoU, built upon WIoUv3, achieved the best performance in terms of mAP0.5 and mAP0.5:0.95, demonstrating superior overall performance.

**Figure 8:**
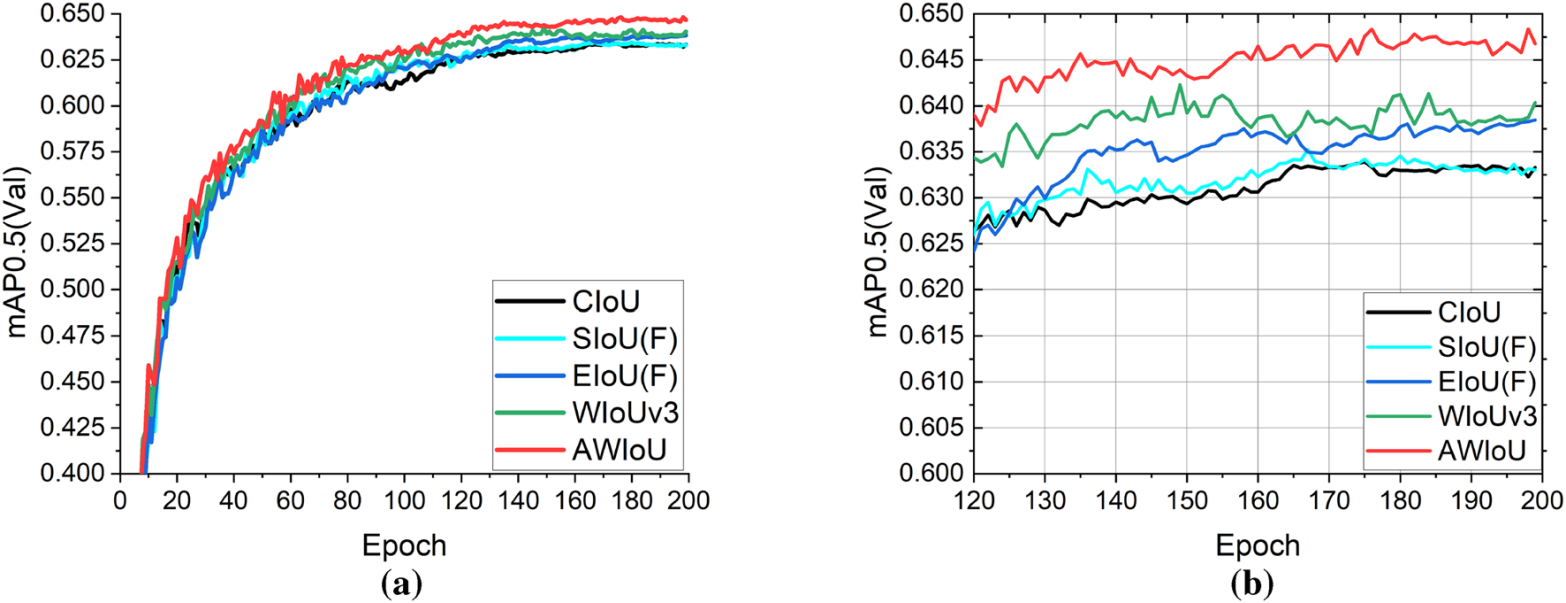
The mAP curve for different IoU in train stage: full process curve (**a**) and curve only from epoch 120 to 200 (**b**).

Next, we analyze the training process and precision performance at different IoU thresholds as the epoch progresses. As depicted in Fig. 8a, the AWIoU curve consistently remained on the outermost side among the five IoU curves, indicating that AWIoU enabled the network architecture to converge faster and achieve higher detection performance. In Fig. 8b, it can be observed that as the epoch approached 130, the curves for all IoU thresholds gradually stabilized. Among them, the curves for CIoU and SIoU(F) were very close, both at the lowest level, while EIoU(F) and WIoUv3 curves were slightly higher, with WIoUv3 demonstrating better performance. The AWIoU curve remained at the highest level, significantly outperforming the second-ranked WIoUv3, thus exhibiting the best detection accuracy.

Hence, both the test results and the analysis of the training process affirm the substantial advantages of the proposed AWIoU in terms of convergence and enhancement in detection accuracy performance. AWIoU proves to be highly suitable for loss evaluation in maritime object detection.

### Ablation experiment

The primary goal of this empirical investigation is to validate the improved performance achieved through alternative optimization components and assess the impact of their integration. Ablation experiments were utilized to analyze these aspects, involving the gradual elimination or addition of specific conditions to evaluate their influence on overall performance. In this study, a progressive optimization approach was employed, where the base model underwent iterative enhancements by sequentially incorporating AWIoU, followed by SMFN, and ultimately GDFP. The performance metrics corresponding to each stage are presented in Table 3.

**Table 3:** Comparation of performance for different improved part with based model YOLOv8n.

Involved component			Metrics				
+ AWIoU	+ SMFN	+ GDFP	mAP0.5	mAP0.5:0.95	PC (M)	FLOPs	FPS
✘	✘	✘	0.623 (0.0%)	0.318 (0.0%)	3.0 (0.0%)	8.1 (0.0%)	98.04 (0.0%)
✔	✘	✘	0.635 (+ 1.9%)	0.321 (+ 0.9%)	3.0 (0.0%)	8.1 (0.0%)	100 (+ 2.0%)
✔	✔	✘	0.671 (+ 7.7%)	**0.358 (+ 12.6%)**	2.3 (− 23.3%)	19.5 (+ 140.7%)	94.34 (− 3.8%)
✔	✔	✔	**0.672 (+ 7.9%)**	0.348 (+ 9.4%)	**0.8 (− 73.3%)**	**6.3 (− 22.2%)**	**101.01 (+ 3.0%)**

The bolded values indicate the optimal performance, while the values in parentheses represent the changes relative to the base model.

Firstly, the introduction of AWIoU did not lead to an improvement in PC performance and FLOPs.However, there was a noticeable increase in mAP0.5 and mAP0.5:0.95, indicating that the improvement brought by AWIoU mainly manifested in terms of detection accuracy and exhibited significant enhancement.Furthermore, although AWIoU did not reduce computational complexity, the adjusted network structure remained efficient, achieving a 2% increase in FPS due to more effective optimization strategies.

After integrating the SMFN module, significant improvements were observed across various performance metrics. Most notably, a 12.6% increase in mAP0.5:0.95 was achieved. Additionally, a substantial 23.3% reduction in PC was attained. However, FLOPs and FPS experienced an increase of 140.7% and a decrease of 3.8% respectively, as the proposed SMFN, despite employing numerous fusion techniques to reduce the parameter count, inevitably led to a significant increase in computational complexity. These results clearly demonstrate the efficacy of integrating SMFN and AWIoU. Furthermore, the implementation of multiple fusion strategies resulted in a notable enhancement in precision performance for object detection, along with a reduction in the parameter count. However, this came at the cost of increased computational work.

Finally, the GDFP lightweight technique, while exhibiting a slight decrease in mAP0.5:0.95 precision growth, was able to compress the base model’s PC by 73.3% and reduce FLOPs by 22.2%, with a 3% increase in FPS. This underscores the potential of integrating GDFP with AWIoU and SMFN, contributing significantly to the model's lightweight design.

In conclusion, the ablation experiment results showcase performance enhancements at each optimization step introduced in the EL-YOLO model compared to the preceding stage. Furthermore, the effective combination of these optimizations demonstrates the feasibility and effectiveness of the proposed improvement approach. Indeed, enhancing detection accuracy and achieving lightweight characteristics for the baseline YOLOv8 model in maritime environments is viable.

### Comparative analysis of object category performance: EL-YOLO vs base model

**Figure 9:**
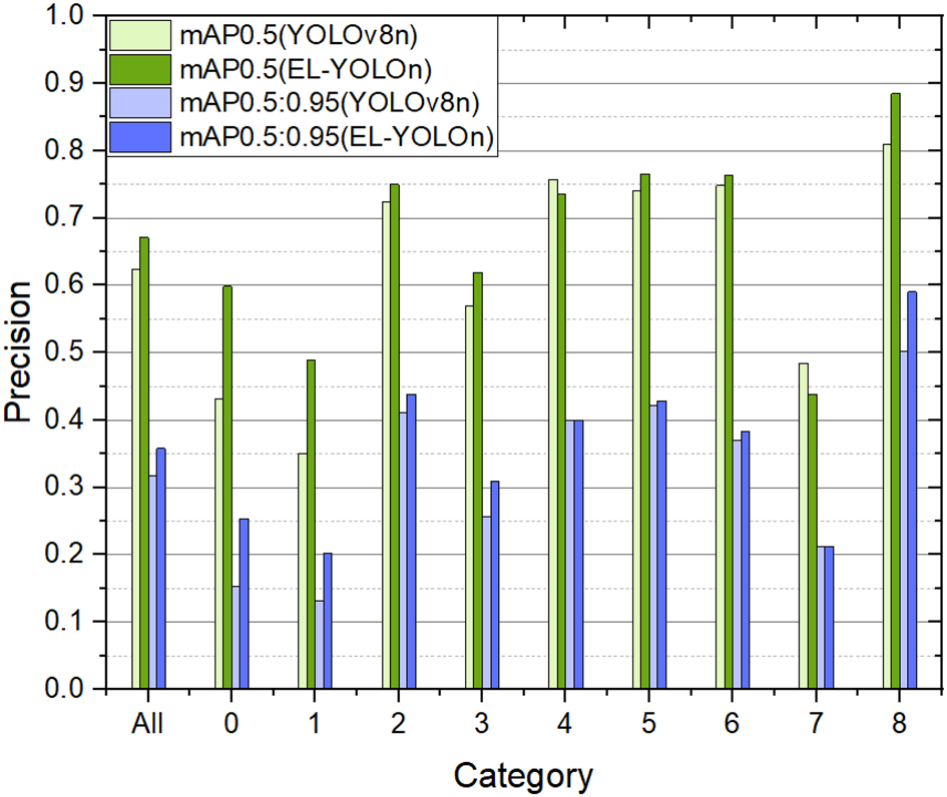
The mAP comparation of YOLOv8n and EL-YOLOn for different categories. Class notes: 0 (Seamark), 1 (Boat), 2 (Sailboat), 3 (Motorboat), 4 (Passenger ship), 5 (Military ship), 6 (Ferry), 7 (Mis boat), 8 (Cruise ship).

Through ablation experiments, we have acquired compelling evidence of the substantial performance enhancement achieved by our proposed EL-YOLO. Looking ahead, our objective is to delve into the accuracy improvement for each object category. As depicted in Fig. 9, with the exception of Class 4 and 7, which experienced a slight decline, all other object classes showcased varying degrees of improvement. Notably, Class 0 and Class 1 exhibited the most significant enhancements, with an increase of 38.74% and 39.42% in mAP0.5, and 66.01% and 53.03% in mAP0.5:0.95, respectively. These two classes, encompassing small or low-quality objects prevalent in maritime scenarios, benefited substantially from the proposed model improvements using AWIoU and SMFN.

Based on the results of this experiment, it is evident that our proposed EL-YOLO demonstrates performance improvements across the majority of object classes, particularly those containing numerous low-quality and small objects. Moreover, as the demand for safety distance and environmental adaptability increases in intelligent ship navigation, the prevalence of these object classes is likely to rise, underscoring the effectiveness of EL-YOLO. Therefore, our proposed EL-YOLO is well-suited for detecting various classes of objects in intelligent maritime applications.

### Comparison with popular realtime models

In the subsequent sections, this paper conducts a comparative analysis between the proposed EL-YOLO model and currently popular models, assessing their respective performances. Notably, two-stage object detection models such as Faster R-CNN^55^ and Mask R-CNN^56^, due to their substantial memory and computational requirements, are omitted from the comparison as they are deemed unsuitable for embedded systems. Similarly, the single-stage SSD^57^ encounters similar challenges. Therefore, for the purpose of comparative experiments, this paper opts to evaluate the widely used YOLO series of models. To ensure fairness, the smallest versions of each series are selected for comparison., For this experiment, two datasets were employed: Seaships (7000) for simple environments and ABOships-9 for complex environments, allowing for a comprehensive comparison.

In the complex environment as shown in Table 4, a similar pattern emerged, where EL-YOLOn exhibited the highest detection accuracy, while YOLOV3-tiny showed the lowest, with mAP0.5 values of 0.672 and 0.615, and mAP0.5:0.95 values of 0.348 and 0.266, respectively. Notably, YOLOv5n and YOLOv7-tiny demonstrated strong performance in terms of mAP0.5 but lagged behind in mAP0.5:0.95, suggesting their proficiency in scenarios where precise localization is less critical. However, these models experienced a notable decline in accuracy when stringent safety requirements and improved localization accuracy were demanded. In regard to FLOPs, YOLOv5n stands out as the smallest, closely followed by our proposed EL-YOLOn. In terms of FPS, our method was second only to YOLOV3-tiny, with a marginal difference between them. In the context of PC, EL-YOLOn once again achieved the best performance.

**Table 4:** Comparison of performance for current popular models in ABOships-9.

Result	Model				
	YOLOV3-tiny^5,8^	YOLOv5n^5,9^	YOLOv7-tiny^60^	YOLOv8n^45^	EL-YOLOn
mAP0.5	0.615	*0.659*	0.658	0.623	**0.672**
mAP0.5:0.95	0.266	0.309	0.311	*0.318*	**0.348**
PC(M)	8.7	*1.8*	6.0	3.0	**0.8**
FLOPs	12.9	**4.2**	13.1	8.1	*6.3*
FPS	**102.04**	100	86.96	98.04	*101.01*

The bolded values indicate the optimal performance, while the italics only values represent the second-best performance.

As depicted in Table 5, in the simple environment, all mainstream models achieved exceptionally high precision in mAP0.5 and mAP0.5:0.95. Notably, the proposed EL-YOLOn attained the top rank in mAP0.5 and the second rank in mAP0.5:0.95, with the latter being very close to the top performer. Additionally, EL-YOLOn exhibited the optimal performance in PC, FPS and FLOPs, benefiting from the removal of unnecessary filters.

**Table 5:** Comparison of performance for current popular models in Seaships (7000).

Result	Model				
	YOLOV3-tiny^5,8^	YOLOv5n^5,9^	YOLOv7-tiny^60^	YOLOv8n^45^	EL-YOLOn
mAP0.5	0.981	0.981	0.981	0.986	**0.988**
mAP0.5:0.95	0.73	0.745	0.742	**0.783**	*0.778*
PC (M)	8.7	*1.8*	6.0	3.0	**0.5**
FLOPs	12.9	*4.2*	13.1	8.1	**4.0**
FPS	*98.03*	90.9	97.08	97.08	**106.38**

The bolded values indicate the optimal performance, while the italics only values represent the second-best performance.

Based on this comparative experiment, it can be unequivocally concluded that the proposed EL-YOLO outperforms state-of-the-art object detection models in terms of mAP0.5 and mAP0.5:0.95 in both simple and complex environments, establishing a significant lead. Simultaneously, EL-YOLO also demonstrated superior lightweight performance, with the only exception being a slightly lower FLOPs and FPS performance in the complex environment, where it ranks second. In all other aspects, EL-YOLO achieved the top rank.

The ABOships and Seaships datasets we utilized are two widely used publicly available datasets, often employed by researchers to validate their detectors. In the subsequent analysis, we compare our results with these datasets. Concerning Seaships, Zhou et al.^61^ proposed an improved model based on YOLOv5, achieving impressive performance with mAP0.5 and mAP0.5:0.95 scores of 0.986 and 0.759, respectively. Regarding two-stage detectors, Shao et al.^10^ validated the optimal performance of ResNet101 with a mAP score of 0.924. Unfortunately, neither study mentioned the experimental results related to PC or FLOPs. Nonetheless, our EL-YOLO achieves outstanding performance with mAP0.5 and mAP0.5:0.95 scores of 0.988 and 0.778, respectively.

For ABOships, Cafaro et al.^62^ employed the real-time detection model YOLOv6n and achieved relatively low performance with mAP0.5 and mAP0.5:0.95 scores of 0.539 and 0.251, respectively. Its performance was even poorer in the case of two-stage detectors, with the best performance of AP 0.352 achieved by Faster RCNN + Inception ResNet V2^11^. Similarly, there was a lack of performance evaluation in terms of lightweight models. Nevertheless, our EL-YOLO achieves leading performance with mAP0.5 and mAP0.5:0.95 scores of 0.672 and 0.348, respectively, retaining its top position.

Therefore, when compared to other models, the proposed EL-YOLO exhibits substantial advantages in terms of detection accuracy and lightweight design.

## Conclusions

To address the challenges of the complex and dynamic maritime environment and achieve efficient object detection with a lightweight network architecture, this paper introduces the EL-YOLO algorithm for RGB camera-based maritime object detection. Extending the capabilities of YOLOv8, EL-YOLO incorporates novel optimizations, namely AWIoU, SMFN, and GDFP, whose effectiveness is experimentally demonstrated through successful integration. The AWIoU optimization focuses on refining the allocation strategy for objects of varying qualities, demonstrating notable advantages in convergence speed and detection accuracy quality when compared to mainstream IoUs. GDFP employees greedily delete filters with lower scores to streamline the network structure, enhancing its efficiency. The newly introduced SMFN enhances feature information comprehension through increased fusion and input–output channels.

Moreover, through a comprehensive comparative analysis, this paper unveils the heightened detection performance of EL-YOLO across diverse maritime object categories. The improvement is particularly conspicuous for categories characterized by numerous low-quality or low-resolution objects, showcasing a robust adaptability to challenging environments. Furthermore, when juxtaposed with other mainstream detection models, EL-YOLO demonstrates exceptional performance in both simple and complex maritime environments, achieving optimal detection accuracy with fewer parameters. Thus, the detailed model analysis and a series of experiments robustly affirm the superiority of the proposed EL-YOLO in intelligent maritime object detection.

The proposed EL-YOLO algorithm emerges as a practical approach producing a balance between lightweight and detection accuracy for maritime object detection. However, some limitations exist. Firstly, due to the lack of fog-related datasets, our algorithm has not considered innovations in defogging, despite fog being a common occurrence in maritime environments. Additionally, we have not accounted for detection under extreme weather conditions such as storms. Nevertheless, our algorithm has achieved superior detection accuracy in both complex and simple environments, while requiring lower computational cost and hardware requirements, which holds significant practical relevance for the widespread adoption of object detection. The introduction of EL-YOLO also provides a direction for future research. By continuously improving and optimizing the model structure, the performance of EL-YOLO on maritime object detection tasks can be further enhanced. In the future, we will continue to investigate ship detection under diverse weather conditions to further improve the model's performance and generalization capability. The streamlined approach of YOLO-based object detection can also be explored for other applications such as in ground autonomous vehicles^63^ and in object detection in tiny objects^64^.

## Data Availability

The datasets in this study is publicly accessible via the links as mentioned in Sect. “Datasets”. For ABOships dataset: https://zenodo.org/records/4736931. For Seaships dataset: http://www.lmars.whu.edu.cn/prof_web/shaozhenfeng/datasets/SeaShips%287000%29.zip.
