# Author Correction: A streamlined approach for intelligent ship object detection using EL-YOLO algorithm

**DOI:** 10.1038/s41598-024-70017-1

**Published:** 2024-08-21

**Authors:** Defu Yang, Mahmud Iwan Solihin, Igi Ardiyanto, Yawen Zhao, Wei Li, Bingyu Cai, Chaoran Chen

**Affiliations:** 1 Faculty of Engineering, Technology and Built Environment, UCSI University, Kuala Lumpur, Malaysia; 2 Department of Electrical and Information Engineering, Faculty of Engineering, Universitas Gadjah Mada, Bulaksumur, Yogyakarta, 55281 Indonesia; 3 School of Computer Science and Technology (School of Software), Guangxi University of Science and Technology, Liuzhou, Guangxi China; 4 School of Advanced Manufacturing, Shantou Polytechnic, Shantou, China

Correction to: *Scientific Reports* 10.1038/s41598-024-64225-y, published online 02 July 2024

The original version of this Article contained errors.

In the ‘NECK’ part of Figure 2, the "CFC" label was incorrectly given as “GBC”. The original Figure 2 and accompanying legend appear below.

**Figure 2:**
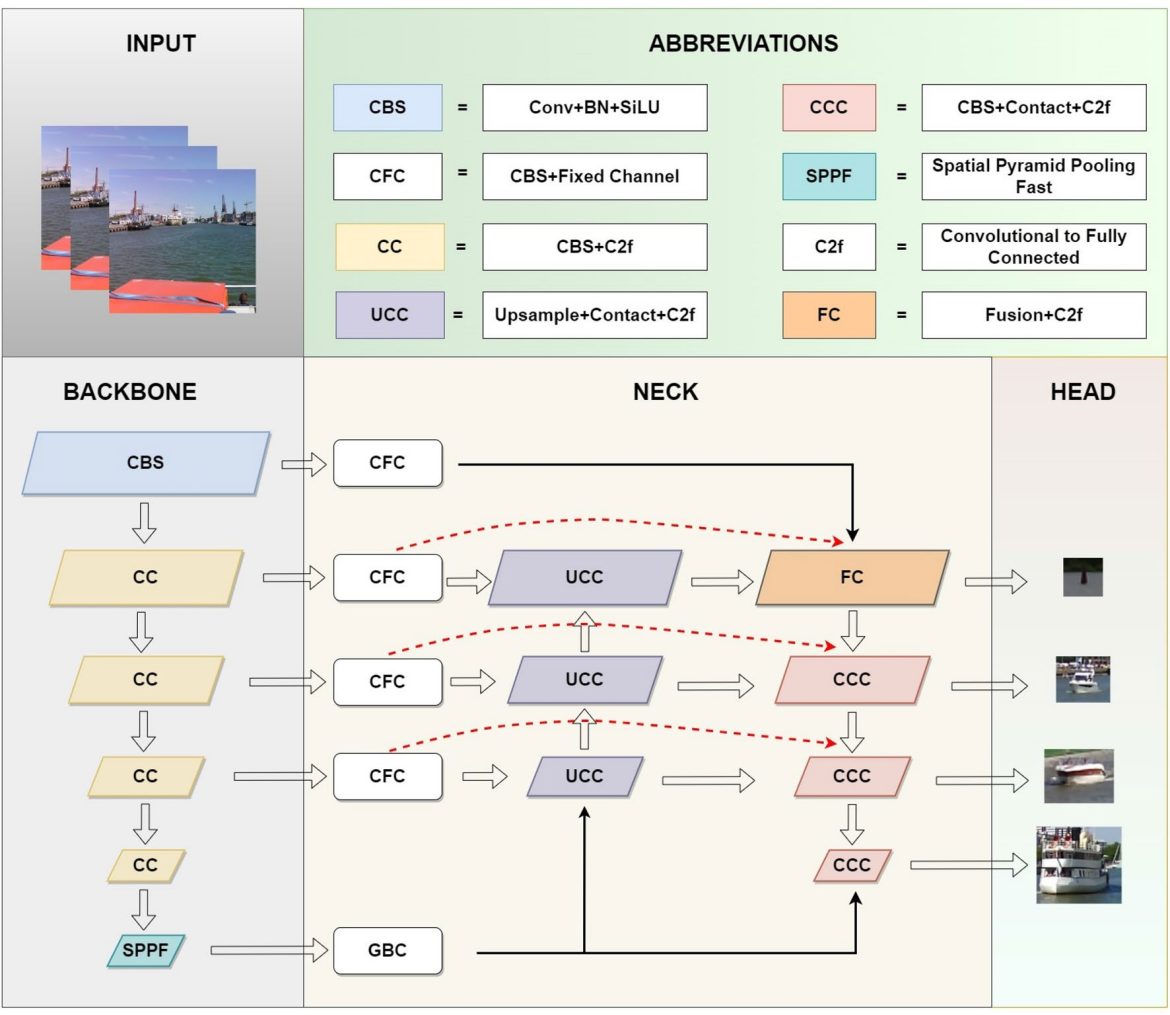
The main network architecture of EL-YOLO (*SPPF* spatial pyramid pooling fast, *C2f* convolutional to fully connected).

In addition, the legend of Figure 8 contained an error, where the “mAP curve” was incorrectly given as “mPA curve”.

The original Article has been corrected.

